# Simultaneous EEG, eye-tracking, behavioral, and screen-capture data during online German language learning

**DOI:** 10.1016/j.dib.2018.11.044

**Published:** 2018-11-13

**Authors:** Gina M. Notaro, Solomon G. Diamond

**Affiliations:** Thayer School of Engineering at Dartmouth, 14 Engineering Drive, Hanover, NH 03755, United States

**Keywords:** Electroencephalography, EEG, Eye-tracking, Mouse-tracking, Duolingo, Naturalistic language learning, Multimodal human data

## Abstract

This article presents concurrent multimodal data, including EEG, eye-tracking, and behavioral data (cursor movements and clicks), acquired from individuals (*N* = 22) while engaging in several German language lessons using the web-based Duolingo interface. Lessons were restricted to visual learning only (excluding audio and speech components), including reading and writing vocabulary words and sentences, and matching vocabulary to images. EEG data was collected using the open-source OpenBCI device utilizing dry Ag-AgCl electrodes, while eye-tracking data was recorded using the Gazepoint GP3 system. Timestamped screen captures associated with mouse click and keypress events and user behavior (cursor movements) were acquired using AutoHotKey macro scripts. These data provide neural (EEG), gaze (eye-tracking), and behavioral (mouse movements, clicks, and keypresses) data, with respect to presented language-learning media (Duolingo screen captures) for a wide range of possible scientific analyses and methods development.

**Specifications table**

**Table:** 

Subject area	*Engineering, Neuroscience, Psychology*
More specific subject area	*Multimodal bio-signals, naturalistic learning, online language learning*
Type of data	*8-channel EEG; eye-tracking; cursor movements, positions and clicks; screen capture images*
How data was acquired	*OpenBCI 32-bit EEG board with Florida Research Instruments dry Ag/AgCl pronged electrodes; Gazepoint GP3 remote eye-tracker; AutoHotKey activity and screen monitoring macro script; German language lessons through the Duolingo web interface on Google Chrome*
Data format	*Raw datasets (.csv and.txt data files,.png screen captures) and processed example (.mat files and.m MATLAB script) provided*
Experimental factors	*Screen capture images containing camera images from the eye-tracker were removed to preserve the anonymity of participants*
Experimental features	*Users wore an EEG headset seated at a computer and completed several lessons on the web-based Duolingo language-learning interface, while also tracking eye movements, screen images, cursor movements, and keypresses*
Data source location	*Hanover, NH, 03755; USA*
Data accessibility	*All data and code provided on FigShare:*https://figshare.com/s/688e387fbfdc000f4e90

**Value of the data**

• The presented dataset could provide a multi-faceted insight into how individuals learn through the web-based language-learning platform Duolingo, from both a neuroscientific and psychological perspective
• This data could be useful to groups developing tools and algorithms for parsing complex behavioral and neuroscientific datasets
• This dataset and described methods provides a starting point for those interested in performing scalable and naturalistic studies utilizing stimuli not generated and controlled by the experimenter (e.g. stimuli on an existing web page)

## 1 Data

The presented data was collected from an integrated low-cost bio-signal setup, containing electroencephalography (EEG), eye-tracking, behavioral (cursor movements and clicks), and screen capture data (generated upon a click or keypress event). Individuals were tasked with completing several German language Duolingo lessons (https://www.duolingo.com/) while these concurrent measures were recorded.

**Fig. 1:**
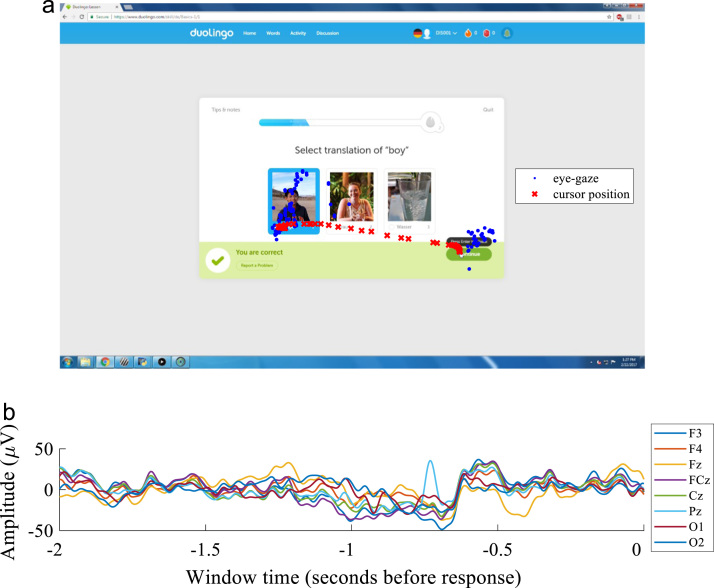
Example data window. (a) A screen capture from one participant (DIS001) correctly answering a German language Duolingo question (Duolingo version February 2017), with overlaid cursor-tracking data (red X׳s) recorded by the AutoHotKey macro script and eye-gaze data (blue dots) recorded by the Gazepoint GP3 eye-tracker, for a window two seconds prior to the displayed correct response submission. (b) Processed OpenBCI EEG data is illustrated for a two-second window prior to the correct response submission in (a). Eight EEG channels (F3, F4, Fz, FCz, Cz, Pz, O1, and O2) were recorded using an elastomeric head mesh and dry Ag-AgCl electrodes.
– from the folder *exampledata*, the following pre-processed.mat files for DIS001 are included to generate an example data window visualization:
  • **headers.mat**: contains header variables for the following data files, described in further detail below
  • **DIS001duoimgdata.mat**: contains ‘duoimgdata’ with timestamp filenames of screen captures (format YYYYMMDDHHMMSSMsMsMs) recorded by the AutoHotKey software, followed by columns marking whether a correct, incorrect, or either response was observed for that screen capture based on the color of the response bar; column headers in variable ‘duoimgdataheader’
  • **DIS001eegdata.mat**: contains ‘eegdata’ with sample indexes, raw 8-channel OpenBCI EEG data at 250 Hz, 3-axis accelerometer data, and a timestamp column converted to millisecond-resolution system time using the raw file date and recorded clock timestamps; column headers in variable ‘eegdataheader’
  • **DIS001eyedata.mat**: contains ‘eyedata’ structure with raw Gazepoint GP3 eye-tracking data at 60 Hz in ‘eyetrack’, with 42 data columns labeled by the variable ‘eyetrackheader’ using variable names from the Gazepoint API manual; also contains calibration data ‘eyecalib’ and ‘eyetotal’ for 9-point and average calibration error, labelled with ‘eyecalibheader’ and ‘eyetotalheader’, respectively
  • **DIS001macrodata.mat**: contains structure ‘macrodata’, with ‘mousetrack’ data labelled with ‘mouseheaders’ corresponding to cursor activity, and ‘clicktimes’ corresponding to the timestamp at which an individual clicked or pressed the enter key (formatted as a matrix with six columns: YYYY MM DD HH MM SS.MsMsMs)
  • **DIS001mouseclickimgtimes.mat**: contains ‘mouseclickimagetimestamps’ containing all system timestamps (millisecond resolution) associated with cursor movements and activity, as well as system timestamps converted from the screen capture image filenames and from the separate file logging the screen capture-generating click and keypress events; useful for computing whether significant lag was present between the click/keypress event and the timestamp at which the screen was recorded (data quality analyses)

Descriptions for the (a) raw and (b) processed data files are as follows:

(a) Raw Data File Descriptions

– **participantnotes.xlsx** provides demographics and experimental notes for all participants, using an anonymized subject code (Duolingo Integrated System = DIS) and number (1–22)
– **DIS0XX.zip** contains each participant׳s complete dataset (with XX as a number between 1 and 22, indicating participant number, with the following subfolders:
  • *ahk*, containing AutoHotKey recorded screen captures with timestamp filenames as YYYYMMDDHHMMSSMsMsMs, providing millisecond resolution timestamp of when the screen capture was recorded; **datetimestamps.txt** corresponding to the timestamps of true click and enter responses (for comparison to screen capture image name timestamps), formatted as YYYY MM DD HH MM SS.MsMsMs; and **timemousetrack.txt**, containing X and Y cursor position (in pixel coordinates) upon mouse movement, whether the left or right mouse button was up or down (U or D), a sampling index, and timestamps (formatted as YYYY MM DD HH MM SS.MsMsMs) of these cursor movements at a max sampling of 60 Hz per second (only recorded upon movement or click events)
  • *eeg*, containing the raw OpenBCI EEG dataset to a 250 Hz sampling rate named with the file date and save time, with (column 1) packet index, (columns 2–9) eight-channels of EEG data (corresponding to 10–20 system electrode locations F3, F4, Fz, FCz, Cz, Pz, O1, and O2), (columns 10–12) three auxiliary (3-axis accelerometer) data columns, and (column 13) clock timestamps with millisecond resolution
  • *eyetrack*, containing **DIS0XX.csv**, Gazepoint GP3 eye-tracking sampled at 60 Hz for each of the 22 participants (XX = 01–22) referencing the top left corner of the screen as [0, 0] and bottom right as [1, 1], and the top row labels corresponding to Gazepoint API (available at www.gazept.com); quality assessment data files **DIS0XX_eyecalib.txt** with the true locations of all nine calibration points and the recorded left- and right- eye positions during each calibration (the bottom row of this file is the most recent calibration, if multiple calibrations were performed), as well as **DIS0XX_eyecalib_error.txt**, containing the number of these eye-tracking calibration points determined to be valid by the tracker and the total error (in pixels) across these points computed by Gazepoint software

(b) Processed Example Data Window File Descriptions

– **exampleduolingoanalysis.m** is the MATLAB code to import data files from one participant, process the data, and plot an example screen capture with overlaid cursor and eye-tracking data and windowed 8-channel EEG data, illustrated in Fig. 1 below

## 2 Experimental design and methods

### 2.1 Participant recruitment and demographics

Twenty-two participants were recruited from Dartmouth College and the surrounding community (mean age: 26.05 ± 5.42 years; 11F, 11M; 21 right-handed, 1 left-handed). All received proper informed consent under an approved Institutional Review Board (IRB) protocol and were required to complete the mini-mental state examination (MMSE) [1] with a passing score of greater than 24 prior to participation to ensure normal neurological function. All participants passed this requirement (mean score: 29.2 ± 0.8). Two participants were wearing glasses during data collection (participants #10 and #11). Datasets were labeled using an anonymized participant code. Participants were screened for their previous language experience. All participants were either native English speakers or fluent in English. Descriptors for each participant, including age, gender, languages spoken, and any additional experimental notes can be found in the file **participantnotes.xlsx**.

### 2.2 Setup and data collection

All data was collected by a Windows 7 desktop computer. Prior to participant arrival, the administrator signed into an anonymized student account, created for each participant using the classrooms feature on the Duolingo site (no personal email was required for these accounts). Permission was acquired from a Duolingo representative to utilize the website as-is for research purposes (site version January-March 2017). The sound output and microphone were then disabled in the Duolingo user account settings to limit the presented material to visual content only, and to prevent artifacts in the EEG data due to muscle movements during speech generation. The sound was also disabled on the recording computer to remove additional possible distractions to the participant.

A 32-bit OpenBCI board (Cyton Biosensing) board connected to a lithium ion polymer rechargeable battery (3.7 V, 500 mAh; Adafruit, New York, NY) was used to acquire the EEG data. Prior to data collection, the FTDI driver latency timer setting used by the OpenBCI device was changed in the Control Panel from the default value of 16 milliseconds to 1 millisecond to allow for accurate recording of data timestamps. The OpenBCI board was connected to Ag-AgCl dry electrodes (Florida Research Instruments, Cocoa Beach, FL) secured within an elastomeric EEG head-cap developed by our lab [2], updated with a more compliant rubber material for increased participant comfort.

Participants were seated at the Windows data acquisition computer for the duration of the task. The elastomeric neoprene EEG head cap was positioned across each participant׳s head at eight standard 10–20 system EEG locations [3]: F3, F4, Fz, FCz, Cz, Pz, O1, and O2. Next, the OpenBCI software GUI was launched (version January 2017) and EEG data collection was initialized. Electrodes were adjusted until an optimal contact and signal quality was achieved (no large amplitude noise or artifacts present). Two dry Ag-AgCl ear clips placed on the left and right ear lobes served as the reference and ground electrodes. To reduce noise, the EEG board was tethered to the desk with the testing computer. Therefore, the 3-axis accelerometer data recorded by the device, output in the data files in addition to the channel data, does not indicate participant movement for this dataset.

An AutoHotKey macro script (AutoHotKey Foundation LLC, Indiana, USA) developed for acquiring user and task data on Windows was also initialized prior to beginning the task. This script was designed to track users’ mouse clicks, enter presses, and cursor movements during the task. Through the macro, screen captures were generated and saved along with the respective system timestamp upon both click events and ‘Enter’ key presses. A separate file logging both screen capture-generating click and ‘Enter’ keypress events was also created. This was designed to match the responses required for interaction and navigation within the Duolingo platform, as answers could be submitted either by pressing the ‘Enter’ key or by clicking the ‘Continue’ button on the screen. The AutoHotKey scripts used to take screen captures (the main file **mouse_screen_capture.ahk** and the module **screen_capture.ahk**) are included with the dataset, along with instructions for use.

Next, the eye-tracker was positioned under the bottom of the computer monitor, flush and continuous with the plane of the screen. The seated distance of the participant to the tracker was adjusted by examining the camera view of the Gazepoint Control software window. A nine-point calibration protocol was then initialized for the Gazepoint GP3 system and calibration metrics recorded. Calibration and data acquisition were initialized through a Python GUI developed for this task. Participants were instructed to stay seated in the same position for the remainder of the task.

Lastly, the anonymized Duolingo account was launched in fullscreen (https://www.duolingo.com/) using a Google Chrome browser window, and task instructions provided verbally to participants. The Duolingo task was displayed on a HP EliteDisplay E221 (Hewlett Packard, Palo Alto, CA, USA) monitor screen (1920 × 1200 resolution, 21.5” diagonal, manufacturer-reported pixel pitch of 0.248 mm). All participants were seated approximately 2 feet from the screen and were instructed to use the keyboard and mouse to submit their responses.

Participants completed a total of four ‘Skills’ using the Duolingo web platform, including ‘Basics 1’, ‘Basics 2’, ‘The’, and ‘Phrases’. These ‘Skills’ contained 10 German lessons. Four main question types were presented to users within the lessons. These included instructions to (i) translate text to or from the foreign language by typing a response (Instructions: ‘Translate this text’), (ii) select a missing word in a sentence (Instructions: ׳Select the missing word׳), (iii) select the appropriate translation of a word by examining images and associated vocabulary (Instructions: ‘Select Translation of [word]’), and (iv) translate a word using the images displayed on the left side of the screen (Instructions: ‘Translate [word]’). All questions were presented within a rectangular region in the center of the screen, with instructions at the top of this rectangle, followed by variable content (e.g., images and vocabulary) depending on the question type, and a bar at the bottom of the rectangle used to both submit responses and indicate response accuracy.

Overall, participants completed all lessons in an average of 20 min, ranging from as little as 7 min for those with prior Duolingo platform experience to 37 min for more deliberate users. At the end of the task, EEG data collection was stopped using the OpenBCI software, while the eye-tracking and macro data was stopped using a Python data collection GUI developed for this task. All data was stored on a secure drive using an anonymized participant code.

### 2.3 Example data windowing and analysis

An example of the data recorded for a single participant (DIS001) was generated for this article using MATLAB R2017a run on a Windows computer (change ‘\’ to ‘/’ for directory names if using a Macintosh computer), according to the following processing steps. All data was first cropped to exclude signals recorded during setup and before the start of the Duolingo task.

#### 2.3.1 Data pre-processing

First, screen captures corresponding to correct and incorrect responses were determined by processing the AutoHotKey screen capture images. These screen captures are of size 1920 × 1200 pixels with the top left corner as x- and y-coordinates [0, 0], respectively, and the bottom right corner as [1920, 1200]. Each screen capture acquired by the AutoHotKey program is associated with a system timestamp, in millisecond resolution, which allows for parsing of all datasets with respect to the task. Screen captures containing non-Duolingo content (e.g. images containing the eye-tracker software camera view) were removed from the dataset to preserve participant anonymity. As the bottom bar on the Duolingo interface changes from gray to green or red upon a correct or incorrect response, respectively, this feature was used to generate an image mask for correct and incorrect responses. These images represented the time point after which a correct or incorrect response was generated by pressing the ‘Check’ answer button (i.e. the screen capture generated when an individual pressed the ‘Continue’ button after submitting and viewing feedback for their response). Therefore, the timestamps prior to the correct and incorrect images were interpreted as the timestamps of response submission.

Second, the EEG data required several stages of processing. As the default OpenBCI EEG software records clock timestamps associated with each line of channel data, the clock timestamps were first converted to system timestamps by utilizing the dates on the data files. EEG data was then filtered using a 3rd order Chebyshev Type II notch filter (20 dB attenuation) from 59–61 Hz to remove powerline noise and a 3rd order, 0.05–20 Hz Butterworth band-pass filter to remove very high and low frequency noise sources. Following this pre-processing, EEG data was parsed into two-second windows prior to submission of a correct answer using the AutoHotKey data.

Next, the X and Y values of one eye-tracking measure (the best-point-of-gaze, or BPOG, metric) were selected for demonstration of the recorded gaze data. Eye-tracking data was first processed by masking with the associated validity metric (BPOGV) output by the device for each line of data. Invalid eye-tracking data points (zeros indicating blinks or missing data, or NaN values) were also excluded. The Gazepoint eye-tracker references [0, 0] as the top left corner of the screen and [1, 1] as the bottom right, and records eye-tracking values within this range to indicate gaze position on the screen. Therefore, eye-tracking data values were converted to the pixel coordinates of the screen capture images (1920 × 1200) for subsequent processing. The eye-tracking data was next parsed into two-second windows immediately prior to answer submission, as processed for the EEG data. Similarly, the cursor data was also parsed into two-second windows prior to each correct response. The X and Y cursor position values were recorded using the same coordinate system as the screen captures (referencing [0, 0] as the top left of the screen and [1920, 1200] as the bottom right); therefore, these values did not need to be converted prior to data windowing.

#### 2.3.2 Combined data example

An example processed data window for a correct Duolingo response is illustrated in Fig. 1 for participant DIS001. Eye-tracking (blue dots) and cursor position (red X׳s) data are overlaid on the screen capture image in panel (a), for the two-second window before the participant submitted a correct response. Similarly, panel (b) shows the filtered 8-channel EEG data (F3, F4, Fz, FCz, Cz, Pz, O1, and O2) recorded using dry Ag-AgCl electrodes for a two-second window prior to this response.

## Funding

Funding for this work was provided by the PhD Innovation Program at the Thayer School of Engineering at Dartmouth College.
